# 24 month outcomes from the Australian National Hand Hygiene Intiative (NHHI)

**DOI:** 10.1186/1753-6561-5-S6-O64

**Published:** 2011-06-29

**Authors:** P Russo, M Cruickshank, ML Grayson

**Affiliations:** 1 Hand Hygiene Australia, Heidlelberg, VIC, Australia; 2 Australian Commission on Safety and Quality in Health Care, Sydney, Australia

## Introduction / objectives

The NHHI was implemented in January 2009 to establish a standardized hand hygiene (HH) culture-change program, including the increased use of alcohol-based handrub (ABHR), throughout all Australian hospitals.

## Methods

A multi-modal culture-change program based on the World Health Organization “5 Moments” program was implemented in all States/Territories, including development of Australian HH guidelines, HHA and State-based healthcare worker (HCW) training program, data collection and analysis tools. Training to standardize HH compliance (HHC) auditing (>90% internal/external validity) was conducted nationally and a 4 monthly data submission schedule established. Electronic and online data submission capability enhanced efficiency and participation. Outcomes 24 months after NHHI commencement were assessed.

## Results

After 24 months, 521 healthcare facilities from all States/Territories submitted HH compliance data, representing approximately 85% and 50% of acute public and private hospital beds, respectively. The overall national (public/private) HHC rate was 68.3% (95%CI: 68.1-68.5%), with State-based rates (public hospitals) of 60.8%>72.6%. National HHC by Moment were: M1: 63.1%, M2: 68.4%, M3: 79.1%, M4: 76.0%, M5: 60.0%, suggesting that education needs to be focused on improvements in HH prior to patient contact, especially before performing procedures (M2). Overall HHC among public hospital medical staff was 53.4% (95%CI: 52.8%>53.9%).

## Conclusion

The NHHI has been associated with a rapid national culture-change among Australian HCWs resulting in significantly improved HHC and a shift to greater use of ABHR. Analysis of NHHI impact on nosocomial disease rates is underway and further improvements in HHC can be expected.

## Disclosure of interest

None declared.

